# microPIR2: a comprehensive database for human–mouse comparative study of microRNA–promoter interactions

**DOI:** 10.1093/database/bau115

**Published:** 2014-11-23

**Authors:** Jittima Piriyapongsa, Chaiwat Bootchai, Chumpol Ngamphiw, Sissades Tongsima

**Affiliations:** Genome Technology Research Unit, National Center for Genetic Engineering and Biotechnology (BIOTEC), National Science and Technology Development Agency (NSTDA), Pathum Thani, Thailand

## Abstract

microRNA (miRNA)–promoter interaction resource (microPIR) is a public database containing over 15 million predicted miRNA target sites located within human promoter sequences. These predicted targets are presented along with their related genomic and experimental data, making the microPIR database the most comprehensive repository of miRNA promoter target sites. Here, we describe major updates of the microPIR database including new target predictions in the mouse genome and revised human target predictions. The updated database (microPIR2) now provides ∼80 million human and 40 million mouse predicted target sites. In addition to being a reference database, microPIR2 is a tool for comparative analysis of target sites on the promoters of human–mouse orthologous genes. In particular, this new feature was designed to identify potential miRNA–promoter interactions conserved between species that could be stronger candidates for further experimental validation. We also incorporated additional supporting information to microPIR2 such as nuclear and cytoplasmic localization of miRNAs and miRNA–disease association. Extra search features were also implemented to enable various investigations of targets of interest.

**Database URL**: http://www4a.biotec.or.th/micropir2

## Background

MicroRNAs (miRNAs) are small regulatory RNAs involved in diverse important biological processes, such as cell development, cell proliferation and differentiation, metabolism, apoptosis and the cell cycle (1–5). In eukaryotes, miRNAs can regulate gene expression by binding to the complementary sites in 3′-UTR sequences of their target genes (2). However, this 3′-UTR binding mechanism is not sufficient to describe all the observed events of miRNA-mediated gene regulation, especially for miRNAs with unidentified target sites. The current paradigm of miRNA function is that they are predominantly located in the cytoplasm where their main role is post-transcriptional control of gene expression by inducing target mRNA degradation, or inhibition of mRNA translation to protein (1, 2). Recently, however, it has been shown that some miRNAs localize predominantly in the cell nucleus, suggesting that they may also regulate genes at the level of transcription by binding to genomic DNA target sites in promoters (6). Furthermore, there are some reports of specific miRNAs controlling gene expression through gene promoter targeting (7–12). Nonetheless, experimental data supporting this novel regulatory mechanism are limited to only a few miRNAs and target genes. A comprehensive database providing predicted miRNA–promoter interactions for the entire genome could assist experimental scientists in exploring interactions of interest and facilitate research in this area.

To address this need, we created the microPIR database with the focus on providing a comprehensive catalogue of all possible miRNA–promoter interactions in the human genome (13). All known experimentally validated supporting data were also recorded along with the predicted target data to add an extra layer of information to the database. To date, microPIR is still the most comprehensive reference on miRNA targets on human genome promoter regions, supporting a different view on how miRNAs regulate genes. After the first release of microPIR, access to the predicted targets on the database has markedly increased. This probably shows the potentially increasing interest in the research of this topic and the potential value of the microPIR database in the future. Given the conservation of miRNAs and associated gene regulatory mechanisms across species, it is plausible that the regulatory role of miRNA through promoter recognition is also present among mammalian species. The major update of microPIR2, thus, includes predicted miRNA promoter targets in mouse genome. Hosting both mouse and human, for which their experimental and other related genomic data are annotated, would allow the comparative analysis of target sites between the two species by our newly developed module, leading to additional useful information for selection of target candidates. Moreover, microPIR2 provides new search features accompanied with extra information in search results, e.g. miRNA–disease association, nuclear vs. cytoplasmic localization of miRNAs, so as to assist generation of new hypotheses related to miRNA promoter targets.

## Construction and Content

**Figure 1.**
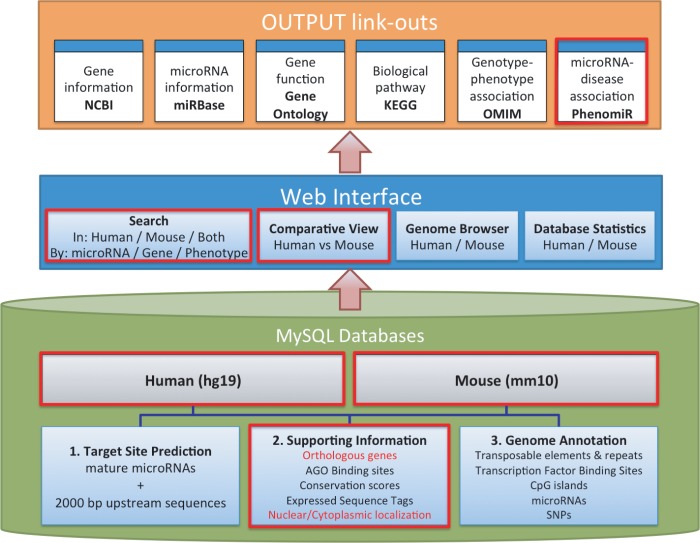
System overview of microPIR2. Three major components of the microPIR2 are demonstrated; MySQL databases, web interface and the output link-outs. The box surrounded by the red line indicates the new/improved features in comparison with microPIR.

The structure of microPIR2 is illustrated in Figure 1. The system is composed of three major parts: (i) MySQL database for hosting miRNA targets and their annotated information, (ii) web interface for displaying miRNA target results and taking in input queries and (iii) the link-outs to other related databases, e.g. gene functions, pathways, etc., for providing extra information related to the miRNA targets in promoter regions.

### MySQL database

Human and mouse databases were separately constructed and they were joined together using human–mouse orthologous gene information from the Mouse Genome Informatics database. Each organism is presented with three types of information, namely predicted promoter target sites, the genomic and experimentally verified data as supporting evidence for the predicted targets, and other genome annotated data so that users can study their relationship with the putative targets. All data were incorporated and managed by MySQL database version 5.5.1.

#### Prediction of miRNA targets

The 2000 bp upstream sequences of all human (hg19 build) and mouse (mm10 build) RefSeq genes were downloaded from the UCSC Table Browser data retrieval tool (14). Human and mouse miRNA data were obtained from the release 19 of miRBase database (15). The computational prediction of miRNA target sites on the upstream promoter sequences was performed using the RNAhybrid program (16). The predicted target sites on both sense and antisense strands must meet the following criteria: perfect complementarity with miRNA seed sequence (positions 2–8) with minimum free energy (MFE) of binding ≤−20 kcal/mol and the target length ≤miRNA length + 10.

#### Data resources

Most of human-related data were obtained from the same source as indicated in the original microPIR publication (13), with all the data updated to the stable build hg19 (at the time of writing). Human SNPs were retrieved from the UCSC Table Browser. Human AGO–DNA interaction data demonstrated by ChIP-seq experiment were obtained from the gene expression omnibus (GEO), GSE40536 experiment of Ago1 ChIP-seq (GSM995943) published by Huang *et al.* (17). The mouse data, based on mouse genome build mm10, including SNPs, expressed sequence tag (EST) sequences, repetitive element sequences and CpG islands, were downloaded from the UCSC Table Browser. The genomic locations of the annotated transcription factor binding site (TFBS) in promoter elements of mouse RefSeq genes were retrieved from ECRBase (18). Argonaute (AGO) binding-site clusters in mouse determined from the mapped CLIP-Seq experimental data were taken from StarBase (19). Human–mouse orthologous gene information was downloaded from the Mouse Genome Informatics database (http://www.informatics.jax.org). Comparative genomic sequence data retrieved from the UCSC Genome Browser (20) were used to assess conservation of the predicted target sites across species. Position-specific conservation scores of mouse mm10 genome version were derived from multiple whole genome sequence alignments between mouse and 59 other vertebrate genomes. For human, the conservation scores obtained from the multiple sequence alignments of 46 vertebrate genomes were used.

### Web interface

Users can query and visualize miRNA targets on promoter regions through the microPIR2 web interface. The user interface comprises four main modules (Figure 1). Human–mouse comparative analysis is the new module added to this version of microPIR database. The search module was also redesigned and added with more features as described below. To interact with the existing modules written in Python scripting language, Python webware (http://www.webwareforpython.org) was used to generate a seamless web interface.

#### The search module

The search module was previously separated in microPIR into basic and advanced search with customized search query. In the new database, users can search for the promoter target sites via two options, the search for miRNA target sites in either human or mouse and the search for targets existing in both organisms for comparative study purpose (Figure 1). For both options, users can customize their miRNA target site parameters.

For the query issued in the single organism option, either an miRNA ID/accession or a target gene ID/symbol is accepted as the input. For the comparative search option, only the ID/symbol of human–mouse orthologous gene is needed. In this update, users can also search by providing disease/phenotype/biological process as input. The association information of the diseases/biological processes with the miRNAs was obtained from PhenomiR database (21). These miRNA–disease data were manually curated and collected from articles that investigated differentially regulated miRNA expression in various diseases and biological processes. The associations between diseases/phenotypes and human target genes were retrieved from the OMIM database (http://www.ncbi.nlm.nih.gov/omim). The microPIR2 database outputs search results by displaying a list of matched target genes and/or miRNAs with detailed information on the corresponding target sites.

To allow large-scale analysis, batch search can be implemented for the search by target gene. Results from this batch search are the list of all miRNAs whose targets are on the queried gene with the details of the corresponding target sites in the promoter. For the single query by individual miRNA, the search result also shows the association information of the queried miRNA with the corresponding diseases and other biological processes as described in the PhenomiR database. In addition, miRNA subcellular localization data obtained from three studies (22–24), which provide the experimentally determined nuclear/cytoplasmic distribution of miRNA, are presented as evidences to support the predicted promoter target sites. The data of experimentally verified binding sites of AGO proteins, which have been reported to be involved in the mechanism of promoter-targeting miRNAs (8, 12), are also incorporated into the database. The presence of AGO binding sites in the proximity of the predicted promoter targets supports the notion that these sites could be targeted by nuclear miRNAs. It should be noted the method used to generate the AGO HITS-CLIP data does not employ steps to separate cytoplasmic from nuclear AGO cross-linked complexes, and so is dominated by the former. However, we assumed that mapped AGO targets in promoter regions arose from the association of nuclear-localized miRNA and AGO directly to promoter DNA, or indirectly to transcribed promoter-associated RNAs (25) that do not make up the body of mature processed mRNA that is exported to the cytoplasm. To better our understanding about these miRNA target sites, microPIR2 graphically visualizes the queried results via genome browser visualizing tool so that users can see the sites and other related information on screen.

#### Human–mouse comparative analysis module

To fully support users’ needs for comparative analysis of human–mouse promoter target sites, a visualization module for human–mouse comparative analysis was developed. The cartoons and diagrams displayed by this module are rendered using scalable vector graphics (SVG). In particular, this module allows users to compare the location of target sites, corresponding miRNA families and the surrounding genomic features along 2000 bp upstream sequence of a human–mouse orthologous gene. This visualization module graphically displays the results of cross-species miRNA target search. For each organism, the target site clusters are displayed along with the clusters of AGO-binding sites, transcription factor binding sites and repetitive sequences. The listing of targeting miRNA families that are either unique or shared between human and mouse orthologous gene is shown along with the links to the details of their target sites. An example of a practical use of this human–mouse comparative analysis module is presented in the Utility section.

### The output link-outs

Each query to microPIR2 will result in listing, graphics and some related web link-outs for additional information of related features, including miRNA information from miRBase and miRNA–disease association as well as bioprocesses from PhenomiR. Resulting genes hosting miRNA targets are presented as the hyperlinks (link-outs) to the National Center for Biotechnology Information (NCBI) Gene database (http://www.ncbi.nlm.nih.gov/gene). Furthermore, we provide more link-outs to their functions, biological pathways and associated diseases published in Gene Ontology (26), KEGG pathway (27) and OMIM databases (http://www.ncbi.nlm.nih.gov/omim), respectively.

## Utility and Discussion

### Main features of microPIR2

Over the past few years, miRNA target gene identification has been one of the main focuses in the study of miRNAs. Still, the mechanisms by which miRNAs regulate gene expression are not completely understood. Our recent microPIR database hosting predicted miRNA targets in promoter regions offers a comprehensive resource for the miRNA research community to quickly generate new hypotheses for an alternate mechanism of miRNA action through gene promoter binding. In this work, we present microPIR2, which does not only come with more updated information but also provides a human–mouse comparative tool for the study of miRNA target evolutionary conservation. The main features (Figure 1) added to microPIR2 are as follows:

1. Predicted miRNA target data of mouse genome as well as the related genomic and experimental information were added.
2. Human–mouse comparative analysis module of miRNA target sites was developed to offer comparisons of promoter targets and their surrounding genomic features between human and mouse orthologous genes.
3. All human data were revised to the current hg19 genome build.
4. Searching for miRNA targets on gene promoter (batch search) was implemented to allow large-scale analysis of target genes.
5. The experimentally determined nuclear/cytoplasmic localization data of miRNA were incorporated as a supporting evidence for promoter-targeting miRNA.
6. The association data between miRNAs and diseases/bioprocesses were included.
7. The new search module was implemented allowing users to query by phenotype/disease of interest.

microPIR2 is built around human genome release 19, in which the number of human mature miRNA sequences has increased to 2042 as compared with 987 miRNAs in release 13 used in the previous release of microPIR. The number of miRNA-promoter predicted interactions found in the human genome has been expanded to about 80 million, approximately five times the number in the original microPIR. In mouse, the number of mature miRNAs is 1281 causing the number of underlying putative promoter interactions to approach 40 million. Both human and mouse have similar number of target sites on both sense and antisense strands.

### The use of comparative analysis module through a case study

The main feature implemented in this database update is the comparative analysis module. The upstream promoter sequences are generally less conserved comparing with gene coding regions (28–30). The presence of predicted binding sites of the same miRNA family on orthologous gene promoters, along with other supporting experimental evidence, provides greater confidence that a biologically functional miRNA target site has been identified.

Here, we demonstrated the human–mouse comparative analysis module for the study of miRNA target sites on their orthologous genes. The comparative analysis helps select new plausible target sites from a pool of predicted targets to be validated experimentally. Given two genes of interest, Protein kinase, AMP-activated, gamma 1 non-catalytic subunit (PRKAG1) and brain-derived neurotrophic factor (BDNF), we wanted to determine for each gene whether there are putative miRNA target sites on both human and mouse promoter sequences.

**Figure 2.**
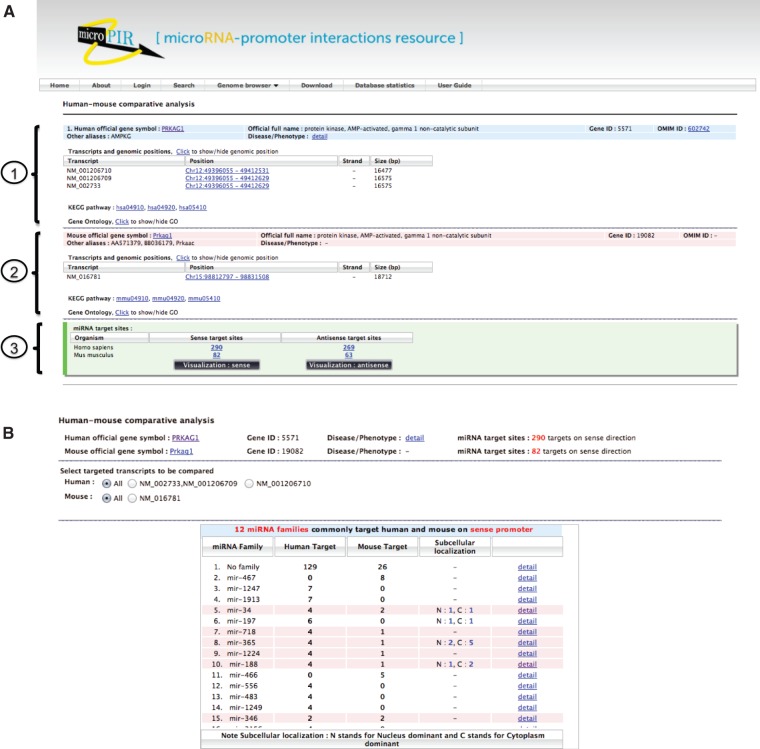
Outputs from comparative search module in microPIR2. (A) The search result of each gene query is grouped into three parts: (1) human gene information, (2) mouse gene information and (3) the summarized numbers of matched target sites. The numbers of predicted target sites found on sense and antisense strands are shown for both organisms. (B) The list of miRNA families, which target the gene query, is displayed in the table with the number of target sites for each family in human and mouse gene. The miRNA families found in common between human and mouse are highlighted in pink color. The ‘Subcellular localization’ column presents the number of miRNA members in each family with the presence of experimental evidence for ‘Nucleus-dominance (N)’ and ‘Cytoplasm-dominance (C)’. The alternative transcript forms of target gene present in human and mouse are listed above the table for users to select for comparison in comparative view module.

To start, the radio button for ‘Human–mouse comparative analysis’ was checked in the *Search* tab, and the search option by ‘Human genes’ was then selected. After entering ‘PRKAG1’ and ‘BDNF’ in the gene list box for batch search, the binding site characteristics were set in the section below by checking the corresponding parameters and/or the appropriate value if required. By clicking on the *Search* button, the results were displayed separately for each gene query. The output showed the detailed information of gene query for both human and mouse; for instance, the official full name and other aliases, gene ID, OMIM ID and related disease/phenotype, the list of transcript isoforms and their genomic positions, associated KEGG pathway and Gene Ontology (Figure 2A). At the bottom panel for each organism, microPIR2 displayed numbers of target sites that comply with the given criteria on sense and antisense strands. Using the default parameter settings (‘upstream region’ 2000 bp, ‘MFE’ ≤ −20 kcal/mol and ‘*p*-value’ ≤ 0.05), 290 and 269 putative target sites were found on sense and antisense strands of human PRKAG1 gene while 82 and 63 sites were found in mouse PRKAG1, respectively. To investigate the resulting target sites further through the new visualization module for human–mouse comparative analysis, we clicked either the *Visualization: sense* or *Visualization: antisense* button for each gene.

**Figure 3.**
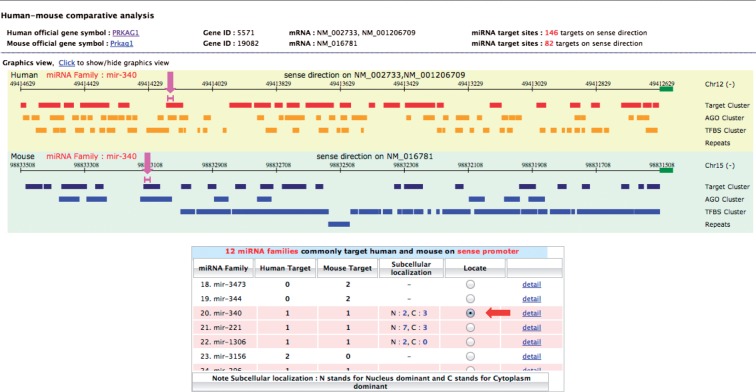
The comparative view of human–mouse miRNA promoter target sites. (A) Graphical view of predicted promoter target sites is illustrated for each organism in separate aligned panels; yellow for human and green for mouse. In each panel, the locations of predicted miRNA target sites and other genomic elements are displayed along 2000 bp sense upsteam sequences of human–mouse orthologous PRKAG1 gene. The genomic features shown include the clusters of AGO binding sites, transcription factor binding sites and repetitive elements. The pink lines, as indicated by the pink arrows, show the positions of target sites of miR-340 family on human and mouse upstream sequences. These are displayed after clicking the radio button in ‘Locate’ column of specified targeting miRNA family as listed in the summarized table (red arrow).

Clicking on the *Visualization: sense* button for PRKAG1 gene, the visualization page showed a table listing all miRNA families targeting the upstream sense sequences of all available PRKAG1 transcript isoforms, along with their target site number in human and mouse genome. The summary results revealed the number of targeting miRNA families found in common between the two organisms. Twelve common families were found in this case as highlighted in Figure 2B. If there are alternative transcripts, users will be prompted to select the gene transcript accessions to be compared between the two species. In the example shown, NM_002733 and NM_016781 accession IDs were selected for human and mouse, respectively, and targets revealed by clicking the *Show target* button. For each organism, the graphical view displays the locations along the upstream sequence of predicted target sites that will be clustered into the same group for the overlapped ones (Figure 3). In addition, we looked at the relationship between target sites and the clusters of AGO binding sites, transcription factor binding sites and repetitive sequences. Such a relationship was displayed in the same panel on two separate tracks. At the bottom of this page, 12 miRNA families, targeting upstream sequences of the selected isoform of PRKAG1 were found in common between the two species. By checking the button in the ‘Locate’ column of each miRNA family, the locations of target sites for this miRNA family were displayed graphically. Users can click on the ‘detail’ link of each miRNA family to see the corresponding information of the relevant target sites. PRKAG1 contains a number of interesting target sites. Out of 12 miRNA families that were predicted to target the PRKAG1 sense promoter in both human and mouse, nine (miR-718, miR-1224, miR-188, miR-346, miR-296, miR-671, miR-221, miR-1306, miR-506) can form highly stable duplex structures with their target sites (MFE ≤ −30 kcal/mol) in both organisms. Some miRNAs in six families (miR-34, miR-188, miR-671, miR-340, miR-221, miR-1306) were shown by at least one experimental study to be nuclear dominant. In addition, six families (miR-34, miR-718, miR-346, miR-671, miR-340, miR-1306) target upstream sequences that contain previously reported AGO binding sites in both organisms. Interestingly, the target sites of all twelve miRNA families are very conserved across vertebrate species with average conservation score ∼≥ 0.9. Hence, these target sites that are conserved between both species and are supported by experimental data could be plausible candidates. In this case, miR-671 and miR-1306 were good candidates for further testing of miRNA–PRKAG1 promoter interactions.

BDNF is a prosurvival factor induced by cortical neurons that is necessary for survival of existing neurons in the brain as well as the growth and differentiation of new neurons and synapses (31, 32). The HuGE Navigator database (33) reports that BDNF is associated with 253 disease terms (MeSH) from 885 publications. BDNF is strongly associated with neuropsychiatric disorders such as schizophrenia (34–37), depression or bipolar disorder (38–41) and Parkinson’s disease (42–45). The pathophysiology and molecular mechanisms of these neurological disorders are poorly understood. There is evidence to suggest that miRNAs play major roles in the expression of genes linked to different neurological diseases (46–51) including BDNF gene (52–55). It is possible that some unidentified miRNAs could regulate these corresponding genes through a novel mechanism. Considering all transcript isoforms, 24 miRNA families in both human and mouse were predicted to bind upstream of BDNF on the sense strand. When considering only the BDNF transcript pair NM_170734 (human) and NM_001048142 (mouse), 11 miRNA shared families were predicted to target the upstream region. Seven out of these 11 families were predicted to bind stably to their target sequences. Experimental evidence for the existence of nuclear miRNAs was also present for the three miRNA families, namely miR-188, miR-671 and miR-30. Target sites of two miRNA families, miR-488 and miR-329, are also very well conserved across both species.

Taking information from the comparative analysis search and the knowledge from the previous studies could help identify interesting target candidates for further experimental testing. For example, members of the miR-30 family were good candidates given that they are predominantly nuclear-localized and were predicted to commonly target human and mouse BDNF sense promoter with strong favorable thermodynamic interaction. One predicted target site of human miR-30c-1-3p also showed the presence of binding sites for AGO2 protein, which was reported to be involved in the miRNA mechanism of promoter binding (12). Members of the miR-30 family were previously reported to target both human and mouse BDNF at the 3′-UTR (55, 56). This observation suggests a possible unidentified interaction between miR-30 and BDNF promoter as well as multiple layers of regulation of BDNF level by targeting both 3′-UTR and promoter regions. By means of the aforementioned procedure, many high potential target candidates could be identified.

## Conclusions

We present microPIR2, which is an updated database of predicted miRNA–promoter interactions in both human and mouse. We implemented the new human–mouse comparative analysis module to help identify common promoter target sites. New search options and a variety of supplementary information such as the nuclear/cytoplasmic localization of miRNA and the reported disease/phenotype related to miRNA or target gene was added to the system. The new microPIR2 database, however, was not designed to host a complete list of verified miRNA promoter target sites. The primary aim is to allow miRNA researchers to investigate multiple layers of information related to miRNA–promoter interactions and identify the potential target candidates that fit with their hypotheses for further experimental studies. Comprehensive exploration of multidimensional of genomic data also facilitates scientists to generate new verifiable ideas and to discover relevant insights into promoter-targeting miRNAs. To keep the predicted targets up-to-date, the database will be updated according to the new version of reference genome data once a year, due to the great burden in computational complexity of these predicted targets.
